# Mercury goes Solid at room temperature at nanoscale and a potential Hg waste storage

**DOI:** 10.1038/s41598-022-06857-6

**Published:** 2022-03-03

**Authors:** N. Kana, R. Morad, M. Akbari, M. Henini, J. Niemela, F. Hacque, A. Gibaud, M. Maaza

**Affiliations:** 1 UNESCO-UNISA-iTLABS/NRF Africa Chair in Nano-Sciences and Nanotechnology, CGS, University of South Africa, Muckleneuk ridge, Pretoria, 0001 South Africa; 2 NANOAFNET, iThemba LABS-National Research Foundation of South Africa, 1 Old Faure Road, Western Cape, 7129 South Africa; 3 Physics and Astronomy Department, Nottingham University, Nottingham, NG7 2RD7 UK; 4 International Centre for Theoretical Physics (ICTP), Str. Costiera, 11, 34151 Trieste, Italy; 5 Physics Department, Rajshahi University, Dakha, Bangladesh; 6 IMMM, UMR 6283 CNRS, University of Le Maine, Bd O. Messiaen, 72085 Le Mans cedex 09, France

**Keywords:** Materials science, Nanoscience and technology, Physics

## Abstract

While room temperature bulk mercury is liquid, it is solid in its nano-configuration (Ø_nano-Hg_ ≤ 2.5 nm). Conjugating the nano-scale size effect and the Laplace driven surface excess pressure, Hg nanoparticles of Ø_nano-Hg_ ≤ 2.4 nm embedded in a 2-D turbostratic Boron Nitride (BN) host matrix exhibited a net crystallization at room temperature via the experimentally observed (101) and (003) diffraction Bragg peaks of the solid Hg rhombohedral α-phase. The observed crystallization is correlated to a surface atomic ordering of 7 to 8 reticular atomic plans of the rhombohedral α-phase. Such a novelty of size effect on phase transition phenomena in Hg is conjugated to a potential Hg waste storage technology. Considering the vapor pressure of bulk Hg, Room Temperature (RT) Solid nano-Hg confinement could represent a potential green approach of Hg waste storage derived from modern halogen efficient light technology.

## Introduction

Mercury (Hg) is among, if not the most peculiar of the periodic elements if one considers its room temperature atypical physical–chemical properties. Mercury was at the pioneering origin of the experimental discovery of superconductivity phenomenon in 1911 by Kamerling Onnes. In high energy physics; its elevated density reduces the physical length of the target and influences the design of the pion capture system, the spread in time of the resulting -burst, as well as the pion production as confirmed in by CERN and Brookhaven National Laboratory using a proton beam of 24 GeV (150 ns). In astronomy, and in regard to its liquid metallic state and therefore its low surface roughness coupled to its high infrared reflectivity, it was used as an efficient large IR liquid mirror as validated by the unit established in New Mexico Observatory. Similarly, its high IR reflecting optical characteristics made it a viable grazing incidence mirror for laser inertial fusion energy experiments as validated by the Lawrence Livermore National Laboratory.

Hg is the unique metal that does not form diatomic molecules in the gas phase. Its bulk room temperature liquid property is correlated to its rare gas-like configuration (Xe) 6s^2^4f^14^d^10^. More accurately, to the relativistic contraction caused by the Dirac dynamics of the valence electrons^1^. As a result of the relativistic mass increase m = m_0_/√(1 − (v/c)), “v/c ~ 0.58”, the radial shrinkage of the effective Bohr radius r_0_ = (ε_0_ h/m_e_ e^2^) of the inner “1s” electrons, is ~ 23%^1^. Since the high order “s” electronic shells have to be orthogonal against the lower ones, they will suffer a similar radius relativistic contraction, inducing a weak coulomb interaction between neighboring Hg atomic sites.

**Figure 1:**
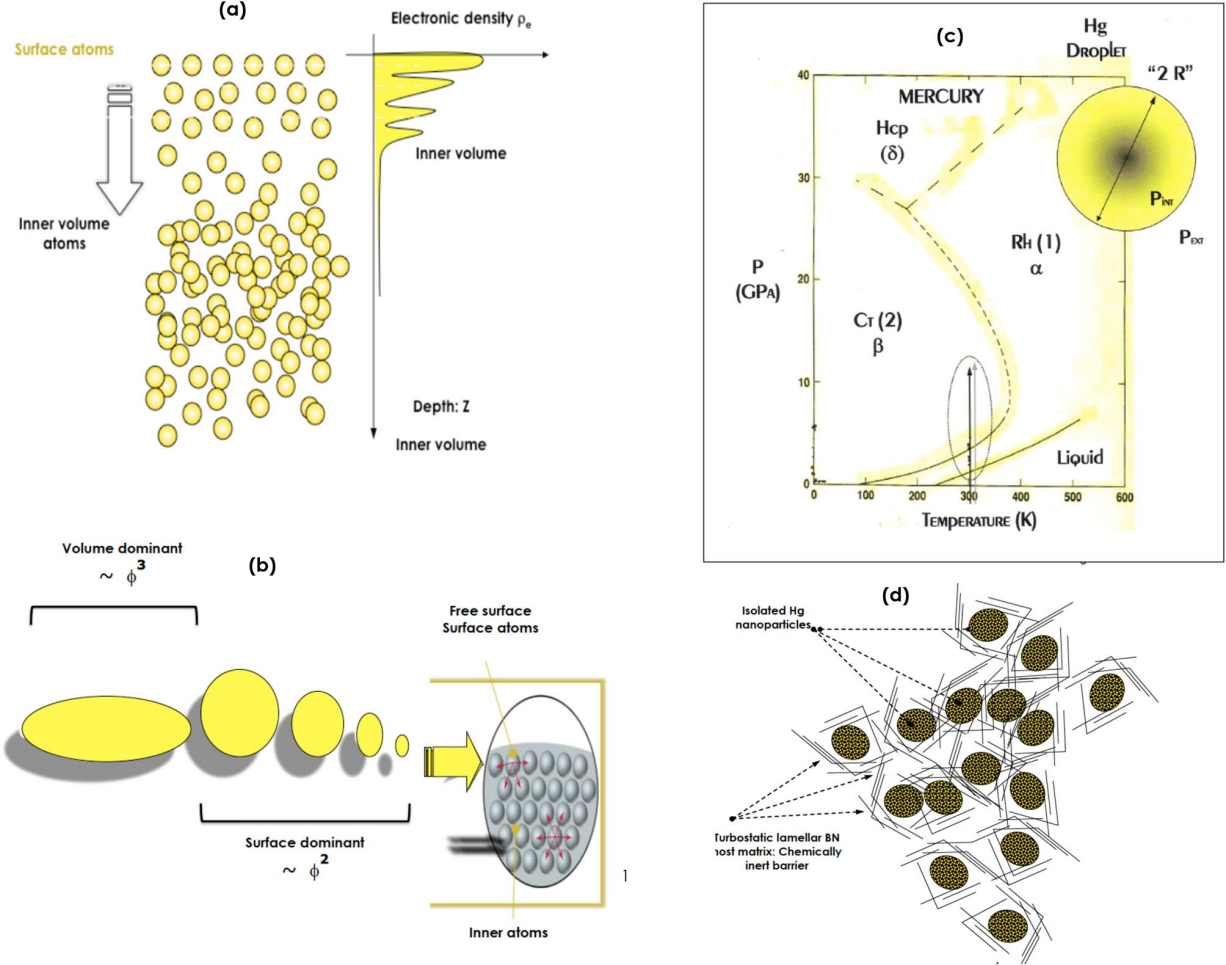
(**a**) Theoretical surface atomic ordering in liquid Hg with the corresponding in depth variation of the electronic density, (**b**) Schematic representation of volume and surface driven shape anisotropy in Hg droplets, (**c**) Hg Phase diagram (according to^11^), (**d**) Schematic representation of the Hg nanoparticles confined in turbostratic BN chemically inert host matrix.

Hg as a singular liquid metal in its bulk form, has the highest elemental surface tension at room temperature; ~ 486 mN/m^2^. The theoretical calculations on liquid–vapor interface of simple metals in general^3,4^ and methods based on the jellium model in particular^5^, and the perturbation expansion up to the second order in the surface “e-ion” pseudo-potential^6,7^, showed that an excessive surface tension could stimulate a significant surface atomic layering of 3–5 atomic planes as depicted in Fig. 1a and the corresponding periodic surface-to volume electron density profile. This surface atomic ordering, in full agreement with capillary wave theory, has been observed by X-ray reflectivity measurements on bulk liquid mercury surface by Pershan et al.^8^. Likewise, Bafile et al.^9,10^ showed that such an atomic ordering was able to be segregated in the bulk liquid mercury by examining the height and the width in addition to the position of the main peaks of the static structure factor S(Q) at ambient conditions. Both X-rays and neutron diffraction S(Q) profiles revealed a structure up to 4–5 discernable peaks: a feature of a local surface atomic ordering^10^.

Such a RT surface atomic ordering observed on flat surface of bulk Hg could be enhanced significantly if not drastically on Hg nano-particles if one could engineer them. Indeed, as a result of their substantial surface/volume ratio, and the 3-D symmetry breakdown, the surface atoms population would be greater in nano-scaled Hg. Henceforth, at such a scale the surface phenomena dominate gravity effects in view of the significantly elevated surface tension of Hg (Fig. 1b). The enhanced surface ratio of nano-scaled Hg of radius “Ø_nano-Hg_/2” should induce an excess of Laplace surface pressure ∆P 4/Ø_nano-Hg_ of tens of MPa. As an estimation, if Ø_nano-Hg_ 2.50 nm, ∆P 0.76 GPa at RT. Considering mercury phase diagram of Fig. 1c, such an excess surface pressure at RT should induce a net crystallization out-of the liquidus space to the solid a-rhombohedric phase^11^ of the nano-Hg (Fig. 1c). Hence, this atomic ordering phenomenon at RT should manifest itself through a significant crystallization out of the liquid phase to the solid rhombohedral “-type” phase.

Consequentially, the originality of this contribution is to validate the room temperature solidification of Hg nanoparticles if their diameter is smaller than the critical value of Ø_nano-Hg_ 2.50 nm. Moreover, under such threshold condition, Hg should exhibit an atomic ordering at room temperature in line with the a-rhombohedral solid phase.

## Experiments, experimental results and discussion

### Synthesis of ultra-small isolated hg nanoparticles below the threshold of Ø_nano-Hg_ 2.5 nm

Apart of the safety aspect, the synthesis of the nano-Hg was by itself an utmost challenge. The considered precursor was mercury (II) acetate Hg (C_2_H_3_O_2_)_2_. However, the foremost additional complexity remains in keeping the nano-Hg separated from each other otherwise the Van der Waals/Otswald ripening type induced agglomeration of the nano-Hg particles will generate larger Hg droplets and hence less surface pressure excess than the required threshold crystallization value of 0.76 GPa at RT. As schematically displayed in Fig. 1d, the 2-D Boron Nitride “BN” isolating host matrix was used to prevent the coalescence process of the nano-Hg once formed. The deliberate choice of such a host matrix is its chemical inertness with Hg and its superior mechanical strength in addition to its 2-D structure.

The ideal precursors for obtaining the BN matrix were Ortho-Boric acid “H_3_BO_3_” and Urea “H_2_NCONH_2_” while Mercury acetate “Hg (C_2_H_3_O_2_)_2_” as the optimal Hg precursor. The chemical reaction taking place was:

$$ {\text{2H}}_{{3}} {\text{BO}}_{{{3} }} + {\text{1H}}_{{2}} {\text{NCONH}}_{{2}} + \xi {\text{Hg }}\left( {{\text{C}}_{{2}} {\text{H}}_{{3}} {\text{O}}_{{2}} } \right)_{{2}} \to \xi {\text{Hg}} + {\text{ 2BN}} + \, \# {\text{ Gases}} $$

While the H_3_BO_3_ and H_2_NCONH_2_ initial compositions were kept stoichiometric, the Hg (C_2_H_3_O_2_)_2_ was varied so to obtain nano-Hg particles within the final BN host matrix. The relative molar initial concentration to BN matrix of Hg (C_2_H_3_O_2_)_2_ was varied accordingly. Smaller is this molar concentration, smaller would be the nano-Hg’s size. The different solutions of H_3_BO_3_, H_2_NCONH_2_ and Hg(C_2_H_3_O_2_)_2_, with the molar fraction of 2,1 and ξ where “ξ” was varied from 1, 1/4 and 1/20 for Hg (C_2_H_3_O_2_)_2_ in de-ionized H_2_O were prepared. The corresponding samples are labeled as: Hg_1/1_-BN, Hg_1/4_-BN, Hg_1/20_-BN. Henceforth, the Hg nanoparticles, if any, would have smaller size in the case of Hg_1/20_-BN. Hence, the focus would be geared mainly on this Hg_1/20_-BN sample.

### Morphology and electron transmission studies

**Figure 2:**
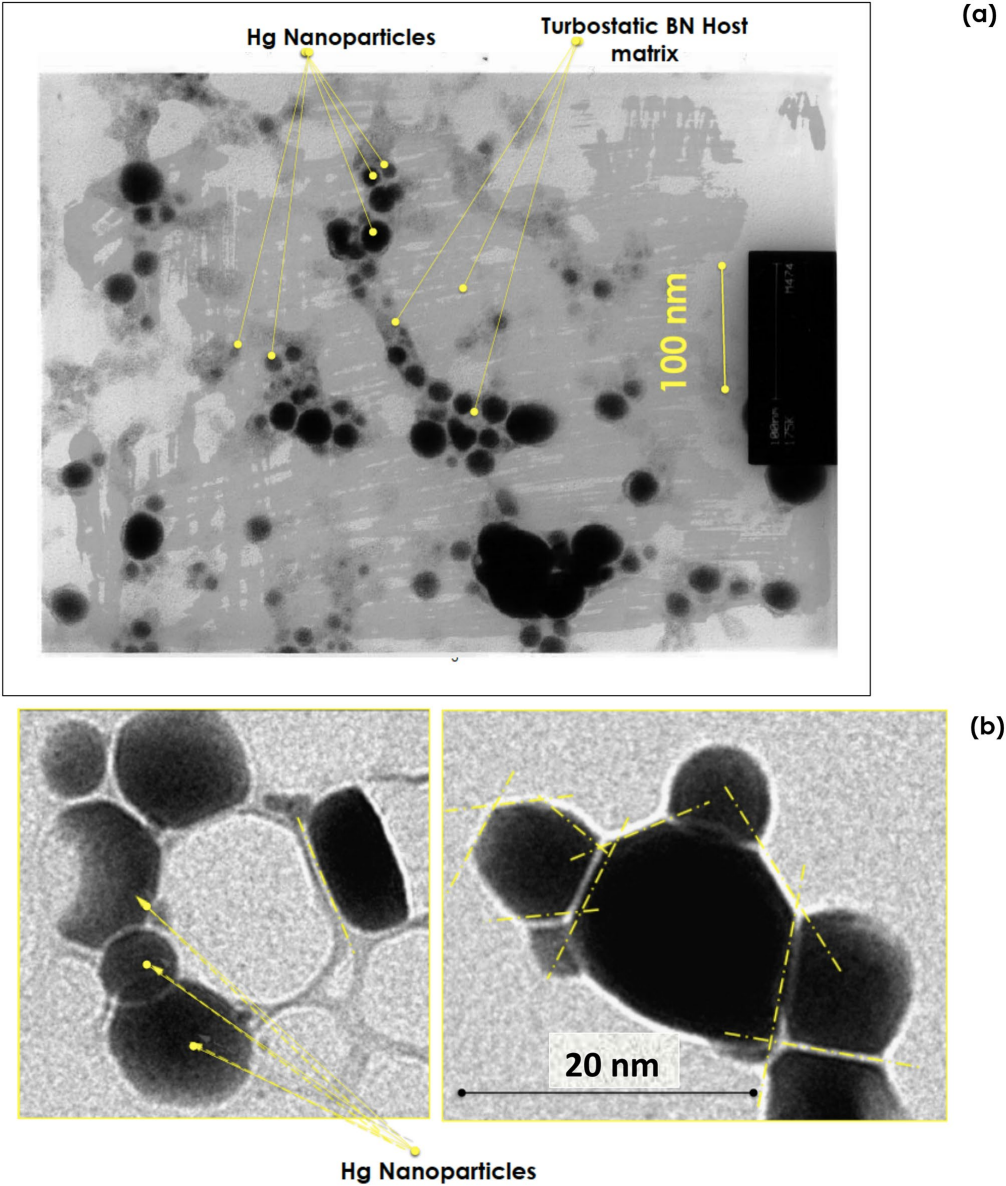
(**a**) Low and (**b**) High magnification Transmission Electron Microscopy of the Hg_1/20_-BN nanocomposite with an early stage average size of the Hg nanoparticles 〈Ø_Hg_〉_TEM_ ~ 2.4 nm dispersed in the turbostratic BN matrix of the Hg_1/20_-BN nanocomposite.

Figure 2a reports a Transmission Electron microscopy (TEM) of the Hg_1/20_-BN nano-composite. The voltage/exposure time have been shortened drastically (≪ 20 s) to minimize the agglomeration of the Hg nanoparticles. The observed rapid coalescence phenomenon during the Transmission Electron microscopy observations Is inherent to the insulating state of the non-percolated Hg-BN nano-composites due to the lack of electrons discharge and heat dissipation caused by the probing electrons beam. Excluding Hg_1/1_-BN sample, the Hg_1/4_—BN and Hg_1/20_–BN nano-composites consisted of nano-sized Hg isolated particles embedded in the BN host matrix. Their average diameter 〈Ø_nano-Hg_〉, at the early stage of the electron beam exposure was estimated to 3.8 and 2.4 nm in Hg_1/4_-BN and Hg_1/20_-BN samples respectively while the Hg_1/1_-BN consisted of relatively significantly large Hg particles; within the submicron range. Subsequent to the heat generated by the TEM electron beam, the primarily well dispersed nano-Hg in Hg_1/4_—BN and Hg_1/20_–BN nano-composites, began to coalesce promptly upon exposition to the electron microscopy beam even if this latter was kept at the minimum voltage possible and an exposure time of 14 s. The TEM pattern of Fig. 2a corresponds to such a final morphological state of Hg_1/20_-BN following a short exposure duration of (~ 14 s). Figure 2b displays a slightly higher magnification but an ultra-short time exposure of Hg_1/20_-BN sample. If the Hg nano-particles are, almost, quasi-spherical in shape with substantially truncated interfaces, the size polydispersity rose promptly subsequent to the slightly higher electrons beam intensity. The new apparent diameter of the Hg nano-particles ranges from 1.5 to 28.9 nm for Hg_1/20_-BN sample. Few larger distorted Hg nanoparticles of ~ 63–70 nm in diameter are observed too. This could be congruent with sample zones which were exposed to a noteworthy heat from the probing e-beam. As highlighted in Fig. 2b, It is worth noting that the sharp interfaces are observed both between Hg-BN as well as Hg-Hg interfaces.

### Crystallographic and phase transition investigations

Thereafter, the Hg_1/ξ_-BN nanocomposites were investigated by XRD. A noteworthy consideration was assigned to the Hg_1/20_-BN nano-composite as the TEM average size of the corresponding nano-Hg, was 〈Ø_Hg_〉_TEM_ ~ 2.4 nm. These latter encaged nano-Hg are undersized sufficiently to undergo the excess of surface pressure above the threshold value of 0.76 GPa and hence would experience a diffraction feature.

**Figure 3:**
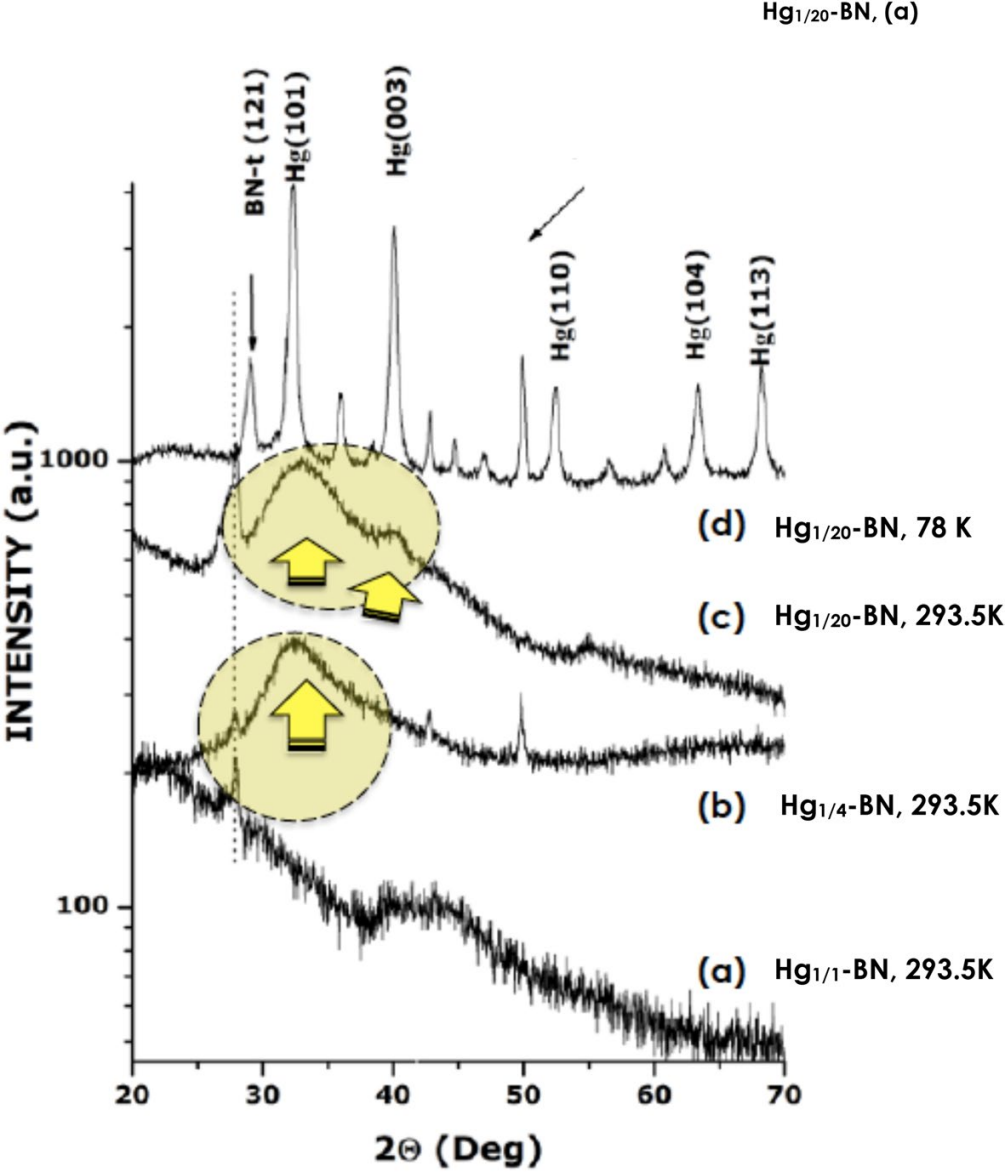
Room temperature X-rays diffraction profiles of the three different samples; (**a**) Hg_1/1_-BN, (**b**) Hg_1/4_-BN, (**c**) Hg_1/20_-BN and (**d**) nitrogen temperature diffraction pattern of this latter sample “Hg_1/20_-BN nano-composite at ~ 78 K.

Figure 3 displays the room temperature XRD profiles of Hg_1/1_-BN (a), Hg_1/4_-BN (b) and Hg_1/20_-BN (c) and the liquid nitrogen (~ 78 K) diffraction pattern of this latter (d) i.e. Hg_1/20_-BN at ~ 78 K. As shown in Fig. 3a, excluding (121) Bragg peak of BN-t host matrix, the highest Hg concentration sample i.e.Hg_1/1_-BN does not exhibit any Bragg peak structure proper to mercury but rather a wide amorphous bump and a very broad peak extending over 10° (40°–50°). These are signatures of an amorphous liquid without any long or mid-range crystalline order^12^.

Figure 3b displays the diffraction pattern of the second lowest Hg concentration i.e. Hg_1/4_-BN. It exhibits 3 narrow diffraction peaks assigned to BN-t host matrix (410), (132) and (203) Bragg peaks “ASTM Card 18-0251” (34). In addition, there is an intense but broad Bragg peak centered at 2Θ ~ 32.72°. This peak with a width at half maximum of ∆Θ ~ 6.3 10^–2^ rad, can be assigned only to crystallized mercury; more precisely to the a-rhombohedral (101) reticular orientation “ASTM Card 09-0253” (35). Comparatively to the diffraction pattern of Hg_1/1_-BN, yet broad, such a Bragg peak could be considered as a signature of a preliminary atomic ordering exhibited mostly by surface mercury atoms within the non-percolated encaged nano-Hg. Using the Scherrer approximation for this Hg (101) broad Bragg peak, the average size of the corresponding Hg nanoparticles is 〈Ø_nano-Hg_〉_S_ ~ 2.4 nm. Likely, such an atomic-like ordering would originate from the surface atoms of the nano-Hg population and those with a smaller diameter according to phase diagram of Fig. 1c.

**Figure 4:**
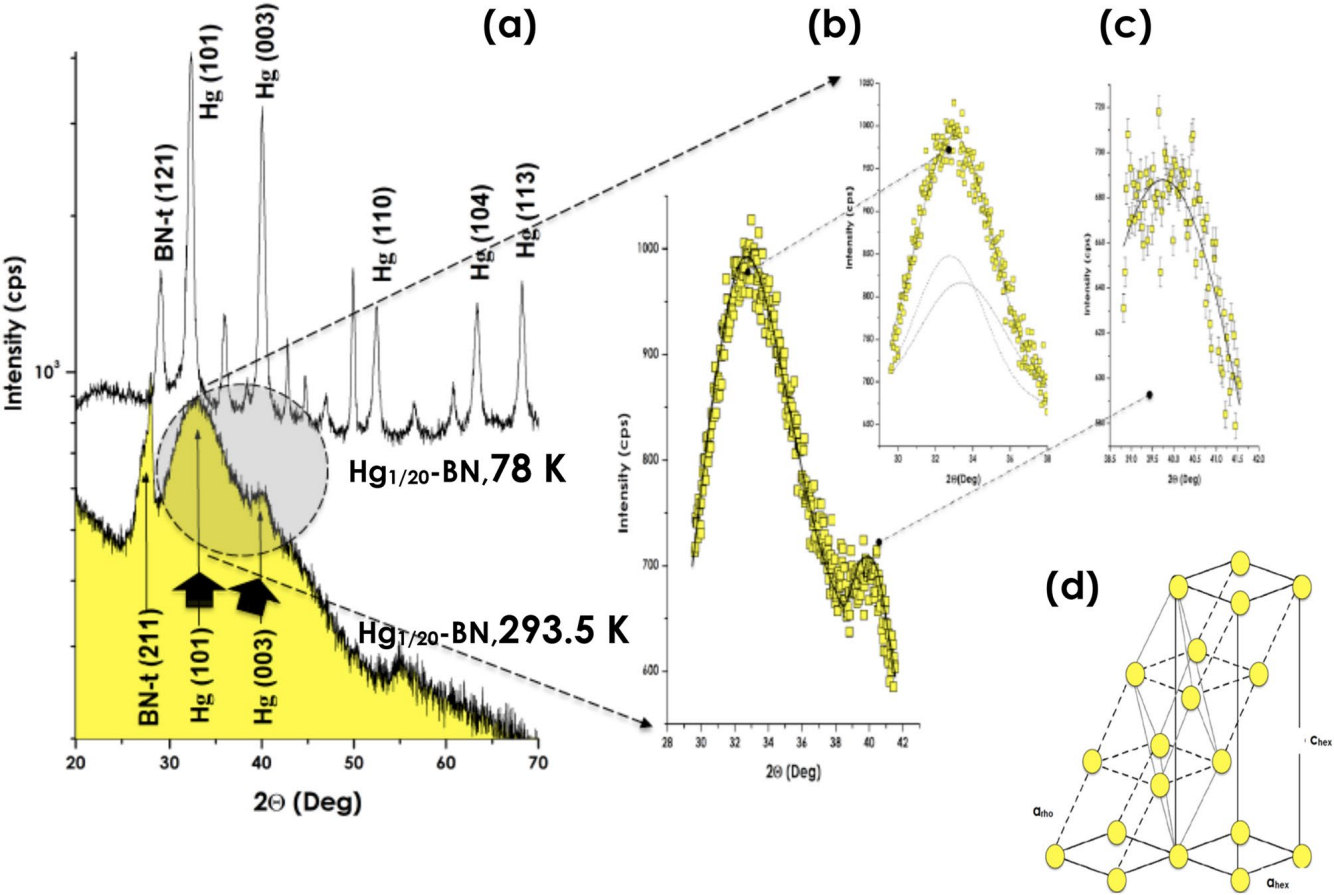
(**a**) Room temperature X-rays diffraction profiles of the Hg_1/20_-BN and its nitrogen temperature. The Inset (**b**,**c**) displays a magnification of the (101) and (003) Bragg peak inferred to (**d**) Solid a-rhombohedric atomic ordering of encaged nano-scaled Hg with 7–8 ordered atomic layers.

To corroborate conclusively the existence of this Hg (101) Bragg peak with the surface atomic layering, the Hg_1/20_-BN nanocomposite was examined extensively both at 293.5 (Fig. 3c) and 78 K (Fig. 3d). As it is the sample with the smallest mercury volume concentration, the corresponding nano-Hg with an average diameter of 〈Ø_nano-Hg_〉_TEM_ ~ 2.4 nm according to the TEM measurements would display the largest surface/volume ratio. The relative Hg (101) intensity should be superior for the same Hg volume concentration. As illustrated in Fig. 3c, not only the relative intensity of the Hg (101) Bragg peak, relatively larger for Hg_1/20_-BN nano-composite, but there is an additional Bragg peak centered at 2Θ ~ 39.7°. Figure 4a and its inset zoom (Fig. 4b,c) focus on Hg_1/20_-BN nanocomposite. This additional diffraction peak has a width at half maximum of ∆Θ ~ 3.580°. Taking into account both its angular position and the relative intensity to the Hg (101) peak and the specific turbostratic structure of the host BN matrix^13^, this second Bragg peak could only be assigned to the 2nd intense crystalline Hg Bragg peak i.e. the Hg (003) crystallographic orientation of the rhombohedral Hg α-phase “ASTM Card 09-0253”. To confirm that the indexed Hg (101) and Hg (003) are proper mercury Bragg peaks originating from the atomically ordered nano-Hg embedded in the BN-t host matrix, the sample Hg _1/20_-BN was cooled to ~ 78.0 K (Fig. 4a). The labeled Hg (101) and Hg (003) develop into sharper peaks with a significant angular shift with 3 new less intense Hg Bragg peaks fitting with Hg(110), Hg(104) and Hg(113) diffraction of solid a-rhombohedral solid Hg in addition to the presence of numerous BN_turbostatic_ diffraction peaks (Fig. 4a and zoom inset). Therefore, the co-existence of the two Bragg peaks, namely, Hg(101) and Hg(003) in the room temperature diffraction pattern of Hg_1/20_-BN nano-composite is the forthright confirmation of the room temperature crystallization of the non-percolated nano-Hg “〈Ø_Hg_〉_TEM_ ~ 2.4 nm” within the BN-t host matrix. These experimental observations, are in support of a surface atomic layering consistent with even 7 to 8 atomic planes ordering (〈a〉 ~ 3.0 Å) as summarized in Fig. 4d. Because the vapor pressure of bulk Hg, embedding Hg in its nano-scaled form in a chemically inert BN matrix could be of a significant advance in the safe storage of Hg and minimization of its hazardous aspect especially the Hg waste derived from modern halogen efficient light technology systems.

In relation to the observed size effect, it is worth mentioning various previous investigations on the confinement of Hg in nanometric configurations. Among them, one should mention the experimental observations in 2003 of Kasperovich et al.^14^, in 1998 of Borisov et al.^15,16^ and in 1986 of Kumzerov et al.^17,18^. More precisely, Kasperovich et al.^14^ have conducted NMR studies on Hg embedded in restricted geometry of nano-porous carbon and nano-porous silica gel with ~ 4.7 and ~ 3.9 nm in radius respectively. The melting—crystallization processes were investigated by measuring the relative integrated intensity of ^199^Hg NMR signals from the liquid phase. Since the integrated intensity of the NMR signal is directly proportional to the amount liquid phase in the sample, the solidification of mercury results in a decrease in the NMR intensity. Similar studies conducted on Hg confined in nano-porous carbon and silica gel showed a decrease of the melting-solidification’s temperature by an amount of 4 to 10 K relatively to the bulk value but no atomic ordering or solidification at room temperature. In the studies of Kumzerov et al. on nano-wires of Hg. The mercury was trapped in natural filamentary nano-systems; chrysotile asbestos Mg_3_Si_2_O_5_(OH)_4_. This natural dielectric host material which exists in the form of regular nano-porous bundles possesses open channels of some tens of nanometers in diameter and lengths of about ~ 1 cm. More precisely, the average diameter of their hollow channels lies within the range of 3.5–15 nm. The conducted studies on mercury nano-wires embedded in these tubular restricted nano-structures showed a clear size effects in transport as well as in superconductivity in addition to melt-crystallization properties but no crystallization at room temperature. As in the case of the previous nano-Hg encaged in carbon and silica nano-pores by Kasperovich et al.^14^, the latter melt-crystallization investigation showed that the corresponding phase transition temperature, however, decreases with size as ∆T = C/〈Ø〉 (41) in a full agreement with the theoretical models so far proposed. Yet again, no atomic ordering/solidification was observed at room temperature of such nano-mercury encaged in the natural restricted geometry of chrysotile asbestos matrix. Borisov et al. conducted ultrasonic studies of the melting-crystallization of mercury encaged in Vycor nano-porous glasses with a pore structure of 7.8 and 12 nm in average size. Once more, it was found that the crystallization temperature changes; More precisely, the crystallization temperature T_crystalization_ was found to vary as ≈ ~ 6 _LS_ T_b_ /L〈Ø _pores_〉 with “_LS_” as the surface energy density in the liquid–solid boundary while “” is the molar volume of the solid phase and “L” is the latent heat with 〈Ø _pores_〉 as the average pore’s size. The ultimate recent literature experimental results to be considered within the framework of this contribution is the investigation by neutron diffraction of the crystallization-melting phase transition of mercury embedded in nano-porous Vycor glass with pores’ size of about 7.0 nm (42). The intensity I_(110)_(T) of the (110) diffraction Bragg peak was followed versus temperature varying from 293.5°K down to ~ 20°K. At room temperature no diffraction Bragg peaks were observed. During the cooling phase, a solidification started only at 205 K resulting in the appearance of (110) Bragg peak. The intensity of the peak was increasing with temperature lowering indicating the growth of the crystalline phase concentration. At T < 100 K the saturation of the (110) intensity was observed. The lower temperature diffraction patterns coincided with the bulk mercury. The measurements in the heating regime have revealed significantly large hysteresis of the I(T) dependency.

In comparison to all above experimental results on nano-Hg embedded in several porous host matrices, albeit it is reduced noticeably, the melting-crystallization temperature of the considered Hg nano-particles is far below room temperature and does not agree with our current observations and those of Magnussen et al., Deutsch et al.^8^. Two conceivable explanations could be advanced: either (i) the size of the concerned Hg nano-particles was not small sufficiently to experience the required excess of surface tension related threshold pressure ∆P of Laplace type of ~ 0.76 GPa “Liquidus” to rhombohedral phase transition as shown in Fig. 3 or/and (ii) the compressibility of the host matrix. Concerning the first size related assumption, it should be noticed that the Hg nano-particles’ size was ranging from 2.5 to 15 nm. Such a size magnitude is, indeed higher than the critical value 〈Ø_(0.76GPa)_〉 of about  2.6 nm. The corresponding ∆P surface Laplace excess pressure are 0.39 and 0.065 GPa for 2.6 to 15 nm respectively. Such values are insufficient to overcome the liquid-rhombohedral frontier of the phase diagram of Fig. 1c which is not the case for the current trapped Hg nano-particles in particular those of Hg_1/20_-BN nano-composite 〈Ø_Hg_〉_TEM_ ~ 2.4 nm, and ∆P 0.76GPa ”. Besides the size effect, the compressibility of the host matrix could be naturally a further component assisting the stability of the observed crystallization of mercury nano-particles. As the BN host matrix is in a turbostratic structural form, its compressibility is the lowest relatively to the considered host matrices such as Vycor glass, polymeric activated carbon or chrysotile asbestos^19,20^. If this is the case, it would be motivating to investigate the Hg_1/20_-BN nano-composite with a diamond anvil cell “DAC” to find out if the rhombohedric -tetragonal phase transition could occur under an external pressure smaller than the required 3.0 GPa at room temperature^21–24^.

As a pre-conclusion, yet size effects were observed in Hg confined in nanometric configurations by Kasperovich et al.^14^, Borisov et al.^15^, Kumzerov et al.^17,18^, no atomic ordering or solidification were observed due to the fact that the minimum restrictive dimensions were in all cases higher than the threshold value of 2.56 nm (Kasperovich (~ 4.7 and ~ 3.9 nm), Borisov (7.8 and 12 nm) and Kumzerov (3.5–15 nm).

### Modelling and computational results

In order to sustain the above experimental observations on atomic ordering/solidification at room temperature of nano-scaled Hg, the density functional theory (DFT) at two levels; atomistic and plane wave with GGA-PBE functionals, including the scalar relativistic effects and dispersion energy, as well as QUANTUM ESPRESSO are used to study the interaction of Hg with the BN surface. The computational calculation presented below and in the Supplementary Section indicates the importance of relativistic effect on the nature and strength of Hg nano-particles adsorption on the BN surface. More precisely, the density functional theory (DFT) calculations were utilized to characterize the bonding of the nano-scaled Hg cluster/surface to the BN surface. The DFT calculation at two levels of theory, atomistic and plane wave, was used by considering the relativistic effect and dispersion correction.

**Figure 5:**
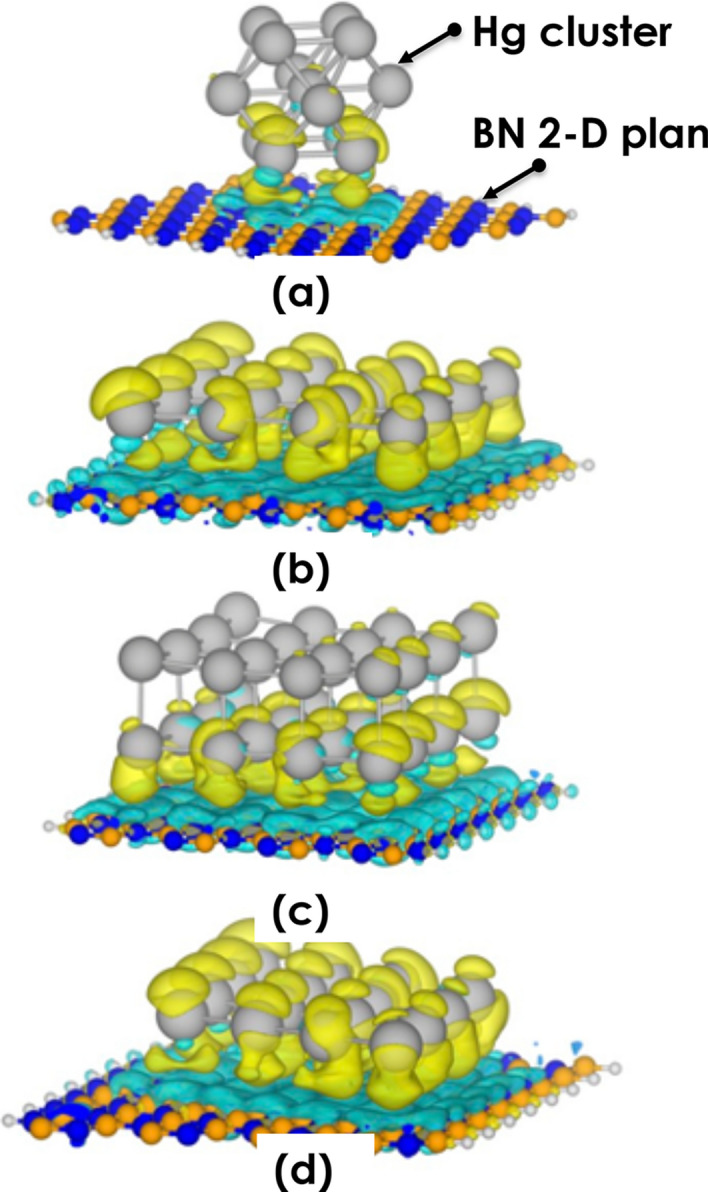
The charge density difference between Mercury and the hBN surface of configuration (**a**) the fcc cluster of Mercury on hBN, (**b**) the Hg (101) surface on hBN surface, (**c**) 2 layers of Hg (101) surface on hBN surface, and (**d**) the Hg (003) surface on hBN surface. The structures are optimized using the plane-wave basis sets at the PBE/D3 level of theory, including the scalar relativistic effect in the pseudopotentials. The iso-value of the charge difference is fixed to 0.0001 e/a.u.^3^. Yellow and blue colors indicate positive and negative levels that correspond to the gain and loss of electrons.

Within the DFT calculations, the Hg cluster with face centered cubic “fcc” structure (Fig. 5a) has been obtained as the stable structure with PBE, BP86, and PW91 functionals^25^. Likewise, the DFT calculations were performed using the Amsterdam Modelling suite of program (ADF)^26^. The generalized gradient approximation (GGA) of Perdew, Burke, and Ernzerhof (PBE)^27^ within the frozen core double-$\zeta$ polarized basis set (DZP) from the ADF basis set library. The influence of relativistic effects has been considered by comparing the non-relativistic (N.R.) and scalar-relativistic (S.R.) ZORA Hamiltonian^5,28^. The dispersion interaction was carried via Grimme DFT-D3 corrections^29,30^.

The adsorption energies were calculated via the generalized gradient approximation (GGA-PBE). The effects of relativistic and dispersion energy corrections were studied. The results seem indicating that the Hg cluster is weakly bonded to the BN surface because the adsorption energies are small (less than ~ 1 eV), which is mainly due to the dispersion interactions. The relativistic effects increase the binding energy of the order of ~ 0.01 eV but change the HOMO–LUMO energy gap significantly.

The fcc cluster of Mercury on the hBN ribbon (Fig. 5b) was studied using Quantum ESPRESSO^31^. There is 10 Å of vacuum in the y–z directions. The GGA-PBE method was utilized to describe the exchange–correlation functional together with the ultra-soft pseudopotential^9,32^ for all atoms. A $4\times 1\times 1$ Monkhorost-Pack mesh grid of k-points was used to sample the Brillouin zone^33–36^. The occupation of electronic states was determined using Gaussian smearing with the width of 0.01 eV, and the real space orbital cut-off of 8.4 Å was conducted.

The charge difference between the mercury cluster, one layer of Hg (101), two layers of Hg (101), and the hBN surface are plotted in Fig. 5. The iso-value of the charge difference is fixed to 0.0001 e a.u.^−3^. Yellow and blue colors indicate positive and negative levels correspond to accumulation and loss of electron charge density upon adsorption of Hg atoms. Changes in the charge density are most pronounced in the case of adsorption of Hg (101) and (003) surfaces, as the interaction energies are also indicating a stronger bond. Generally, the accumulation of charge is mostly around the Hg atoms.

From technological applications viewpoint, yet not presented by say, and in view of the theoretical and the experimental obtained results, it might be secure enough to propose this approach as a potential way of storing Hg at room temperature. The solid aspect of nano-scaled Hg minimizes its vapor hazard at room temperature and hence the idea of safe storage.

## Conclusions

A size effect in nano-scaled Hg dispersed in a 2-D BN host matrix was observed at room temperature. For Hg nanoparticles with a diameter smaller than the threshold value of 2.5 nm as defined by the P–T phase diagram, exhibit a net crystallization manifesting itself through surface atomic layering of about 7–8 atomic layers. Below such a threshold value of 2.5 nm, Hg is solid at room temperature with an a-rhombohedral crystallographic structure with an average lattice parameter 〈a〉 ~ 3.005 Å. The theoretical modelling showed using various codes and approximations indicated, each and all, a crystal-clear accumulation and loss of electron charge density upon adsorption of Hg atoms. The changes in the charge density are most pronounced in the case of adsorption of Hg (101) and (003) surfaces. This latter is in support of the experimentally observed atomic ordering /solidification of nano-scaled Hg at room temperature. Considering the vapor pressure of liquid bulk Hg, embedding Hg in its nano-scaled form in a chemically inert BN matrices could be of a significant advance in the safe storage of Hg and minimization of its hazardous aspect especially the Hg waste derived from modern halogen efficient light systems. As a follow up of this fundamental study, is to carry out synchrotron-based techniques such as EXAFS, SAXS and powder XRD.

## Supplementary Information

Supplementary Information.

## References

[1] Pyykkö P (1978). Relativistic effects in structural chemistry. Adv. Quantum Chem..

[2] Wilkinson MC (1972). Surface properties of mercury. Chem. Rev..

[3] Evans R (1974). The Monte Carlo method for the study of phase transitions: A review of some recent progress. J. Phys. C..

[4] Amokrane S (1982). A pseudo-atom theory for the liquid–vapor interface of simple metals. J. Phys. Chem..

[5] Lang ND (1970). Theory of metal surfaces. Phys. Rev. B.

[6] Chacon E (1985). Nonlocal kinetic energy functional for nonhomogeneous electron systems. Phys. Rev. B..

[7] Gomez MA (1992). ” Electronic structure: Wide-band, narrow-band, and strongly correlated systems. Phys. Rev. B.

[8] Magnussen M (1995). X-ray reflectivity measurements of surface layering in liquid mercury. Phys. Rev. Lett..

[9] Bafile U (1999). Ab initio molecular dynamics study of the static, dynamic, and electronic properties of liquid mercury at room temperature. J. Non-Cryst. Solids..

[10] Bafile U (2000). The microscopic structure of liquid mercury from neutron and X-ray diffraction. Physica. B.

[11] Young B (1992). Phase Diagram of Elements.

[12] Gaston N (2006). The lattice structure of mercury: Influence of electronic correlation. Phys. Rev. B.

[13] Matsui T (2003). Synthesis and characterization of cerium oxide nanoparticles coated with turbostratic boron nitride. J. Mater. Chem..

[14] Kasperovich VS, Charnaya EV, Tien C, Wur CS (2003). NMR of mercury in porous carbon and silica gel. Phys. Solid State.

[15] Borisov BF (1998). Solidification and melting of mercury in a porous glass as studied by NMR and acoustic techniques. Phys. Rev. B..

[16] Michel D, Borisov BF, Charnaya EV (1999). Solidification and melting of gallium and mercury in porous glasses as studied by NMR and acoustic techniques. Nanostruct. Mater..

[17] Kumzerov, Y. A. In *Electronic Properties Of Near-One Dimensional Metallic Wires*. PhD Thesis, Leningrad (1986).

[18] Kumzerov YA (1997). Proceedings of the WTEC Workshop On Russian Research and Development Activities on Nanoparticles and Nanostructured Materials.

[19] Barret JS (1957). The structure of mercury at low temperatures. Acta Crystallogr..

[20] Christenson HK, Phys J (2001). Confinement effects on freezing and melting. Condens. Matter..

[21] Vakhrushev SB, Kumzerov YuA, Nabereznov AA (1995). Freezing and melting of mercury in porous glass. Phys. Rev. B..

[22] Donohue J (1974). The Structure of the Elements.

[23] Moriarty JA (1982). Density-functional formulation of the generalized pseudopotential theory. II. Phys. Rev. B..

[24] Solozehenko VL, Solozhenko EG (2001). Equation of state of turbostratic boron nitride. High Pressure Res..

[25] Gaston N, Paulus B, Rosciszewski K, Schwerdtfeger P, Stoll H (2006). Lattice structure of mercury: Influence of electronic correlation. Phys. Rev. B.

[26] ADF2019. *01, Theoretical Chemistry*. V. U., SCM. http://www.scm.com.

[27] Perdew P, Burke K, Ernzerhof M (1996). Generalized gradient approximation made simple. Phys. Rev. Lett..

[28] Van Lenthe E, Baerends EJ, Snijders JG (1994). Relativistic total energy using regular approximations. J. Chem. Phys..

[29] Grimme S, Antony J, Ehrlich S, Krieg H (2010). A consistent and accurate ab initio parametrization of density functional dispersion correction (DFT-D) for the 94 elements Pu. J. Chem. Phys..

[30] Grimme S, Ehrlich S, Goerigk L (2011). Effect of the damping function in dispersion corrected density functional theory. J. Comput. Chem..

[31] Scandolo S, Giannozzi P, Cavazzoni C, de Gironcoli S, Pasquarello A, Baroni S (2005). First-principles codes for computational crystallography in the Quantum-ESPRESSO package. Z. Kristallogr..

[32] Lejaeghere K (2016). Reproducibility in density functional theory calculations of solids. Science.

[33] Monkhorst HJ, Pack JD (1976). Special points for Brillouin-zone integrations. Phys. Rev. B.

[34] Singh PP (1994). From hexagonal close packed to rhombohedral structure: Relativistic effects in Zn, Cd, and Hg. Phys. Rev. Lett..

[35] Kim WY, Nautiyal T, Youn SJ, Kim KS (2005). Anomalous behavior of Mercury in one dimension: Density-functional calculations. Phys. Rev. B.

[36] Paulus B, Rosciszewski K (2004). A highly accurate potential energy curve for the mercury dimer. Chem. Phys. Lett..

