# Supplementary material for: Mercury goes Solid at room temperature at nanoscale and a potential Hg waste storage
Source: Sci Rep. 2022 Mar 3;12:3494. doi: 10.1038/s41598-022-06857-6 (PMC8894422; doi:10.1038/s41598-022-06857-6)
Supplement: Supplementary file 1 — Supplementary Information. [file 41598_2022_6857_MOESM1_ESM.docx]

**ADDITIONAL INFORMATION**

**Mercury goes Solid at room temperature at nanoscale & a potential Hg waste storage**

**S1. Synthesis of Hg Nanoparticles & their space confinement in BN host matrix**

The considered precursor was mercury (II) acetate Hg (C_2_H_3_O_2_)_2_ while Ortho-Boric acid “H_3_BO_3_” and Urea “H_2_NCONH_2_” were considered as a source for the host matrix of turbostratic BN. The chemical reaction taking place was: 2H_3_BO_3_ + 1H_2_NCONH_2_ + ξ Hg (C_2_H_3_O_2_)_2_ →ξHg + 2BN + # Gases. While the H_3_BO_3_ and H_2_NCONH_2_ initial compositions were kept stoichiometric, the Hg (C_2_H_3_O_2_)_2_ was varied so to obtain nano-Hg particles within the final BN host matrix. The relative molar initial concentration to BN matrix of Hg (C_2_H_3_O_2_)_2_ was varied accordingly. Smaller is this molar concentration, smaller would be the nano-Hg’s size. The different solutions of H_3_BO_3_, H_2_NCONH_2_ and Hg(C_2_H_3_O_2_)_2_ , with the molar fraction of 2,1 and ξ where “ξ” was varied from 1, 1/4 and 1/20 for Hg (C_2_H_3_O_2_)_2_ in de-ionized H_2_O were prepared at room temperature. The corresponding samples are labeled as: Hg_1/1_-BN, Hg_1/4_-BN, Hg_1/20_-BN.

Following a thorough homogeneous steering, the solutions were sprayed individually in liquid Nitrogen through a nozzle of ≈1μm in diameter under an external pressure of Nitrogen carrier gas. The entire spray process took place for about 5 to 10 min for each experiment. The 3 different frozen solutions consisted of white ultra-porous framework. Each open frozen arrangement was transferred to a standard freeze-drying system where it went through a gradual sublimation phenomenon from ≈78°K to 298°K in vacuum. During such a gradual stage, the solid solvent “solid H_2_O” was taken off through a sublimation process. The final dried product consisted of an homogeneous mixture of the 3 initial salts of H_3_BO_3_, H_2_NCONH_2_ & Hg(C_2_H_3_O_2_)_2_ but in an ultra-porous form. Before the decomposition phase to obtain Hg particles embedded in BN host matrix, Differential Scanning Calorimetry “DSC” as well as thermogravimetry analysis were conducted (Fig.S.1.). The DSC experiments were carried out in the temperature range of 25-400°C under pure Argon gas flow to locate the optimal temperature of decomposition of the triple precursor ultra-porous powders so as to minimize the temperature of decomposition in the furnace. Fig.S.1 shows the corresponding DSC profiles of the 3 original precursors labeled as Hg_ξ_-BN with ξ =1/1,1/4 and 1/20. They exhibit mainly the typical different endothermic peaks of the chemical reaction of H_3_BO_3_ and H_2_NCONH_2_ to form the BN host matrix as well as the decomposition of the Hg(C_2_H_3_O_2_)_2_. Taking into account the thermogravimetry analysis and the present DSC measurements, the temperature corresponding to full decomposition was fixed at approximately ~250°C. The decomposition of the 3 ultra-porous precursors was performed by placing them in a quartz boat placed in a sealed quartz tube placed in a furnace under a continuous flow of pure hydrogen “99.997%, Afrox”. The pure hydrogen gas was considered to ensure the non-oxidation of the mercury nano-particles once formed within the BN host matrix. While the optimized decomposition period was shortened to less than 10 seconds, the final decomposed products were submitted to an instantaneous quenching process so to minimize the coalescence phenomenon of the formed mercury nano-particles within the BN host matrix.

**Fig.S.1:** Differential Scanning Calorimetry profiles of the 3 different samples; Hg_1/1_-BN, Hg_1/4_-BN, andHg_1/20_-BN.

**S2. Transmission electron microscopy**

The transmission electron microscopy were conducted on a Zeiss 912-Omega transmission electron microscope is equipped with an in-line Omega filter for composition selective transmission imaging and quantitative electron diffraction. This moderate resolution (0.35nm) microscope has a liquid nitrogen-cooled double-tilt specimen holder, is capable of hollow-cone illumination for dark-field imaging, and is equipped with a slow-scan CCD camera for direct digital image acquisition. The **point resolution, voltage** and **magnification are:** TEM 0.34 nm, 60-120 kV and 80-500000x respectively. The TEM observations were limited to a minimal exposure time of 14s.

**Fig.S.2:** Size distribution of the Hg Nanoparticles dispersed within the BN host matrix (sample Hg_1/20_-BN at Room Temperature )once illuminated with the TEM electron beam during a 14 seconds time exposure (Deduced from the size data analysis of several TEM images similar to Fig.2.a).

**S3. X rays diffraction**

The X-ray diffraction measurements were carried out on a standard Brucker type X rays diffraction unit with a Ni-filtered Cu_Kα1_ = 1.545Å” over the angular range of 2Θ of 10-80 Deg. The total accumulation time was identical for all samples “≈7h30min”.

**
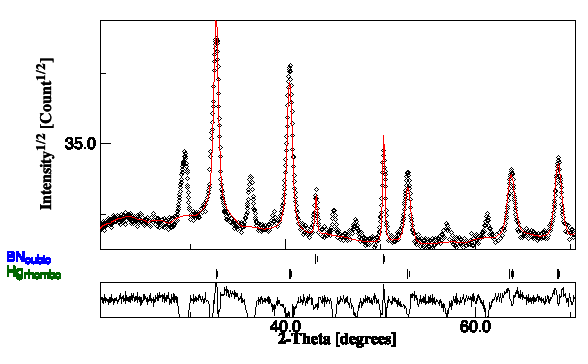
**

**Fig.S.3:** XRD spectrum of the Hg_1/20_-BN at N_2_ Temperature with its MAUD Rietveld treated profile.

As per Table 1, we definitely identified all the Bragg reflections (Fig.S.3) of solid mercury having the rhombohedral symmetry belonging to the R-3r space group. A fit to the observed data yielded a lattice parameter a_r_ = 0.2987±0.0001nm and an alpha angle α=70.76±0.02°. As summarized in Table 1, the observed 7 Bragg peaks of the sample Hg_1/20_-BN at N_2_ sample correspond to the rhombohedric crystalline phase of Solid Hg.

| **Hg Bragg Peak** | **h** | **k** | **l** | **2Θ (Deg)** | **d_hkl_ (Å)** |
| --- | --- | --- | --- | --- | --- |
| 1 | 1 | 0 | 0 | 32.66 | 2.723 |
| 2 | 1 | 1 | 0 | 40.40 | 2.228 |
| 3 | 1 | 1 | 1 | 40.58 | 2.221 |
| 4 | -1 | 1 | 0 | 52.78 | 1.729 |
| 5 | -1 | 1 | 1 | 63.56 | 1.462 |
| 6 | 2 | 0 | 0 | 63.95 | 1.456 |
| 7 | 1 | 2 | 0 | 68.71 | 1.366 |

**Table 1 :** Miller indices, 2θ positions and d_hkl_ of the seven first Bragg reflections of solid mercury observed in the X-ray data set corresponding to sample Hg_1/20_-BN at N_2_ temperature.

The analysis of the peaks broadening yielded a size of coherently diffracting crystallites L=33±3nm with a strain parameter s = 0.0039±0.0003 as per the MAUD Rietveld treatment [1]. The other non indexed Bragg peak are indubitably related to the BN host matrix as BN is chemically stable & does not react with Hg.

Likewise, the MAUD & Rietveld treatment was carried out on the XRD profiles of the Room Temperatures of the Hg_1/4_-BN & Hg_1/20_-BN samples (Fig.S.4 & Fig.S.5). The RT X-ray patterns of the Hg_1/4_-BN & Hg_1/20_-BN scattering pattern assumes the presence of solid mercury (see Fig.S.4 & Fig.S.5). The relatively sharp reflections were observed and are attributed to the host matrix of BN.

In addition, two peaks located at 2θ=43.23° and 2θ=50.34° were attributed to the (111) and (200) reflections of cubic boron nitride (space group F-43m) with a lattice parameter a=0.3621nm. A preferred orientation using the March-Dollase on the 200 Bragg reflection was introduced.

The calculation in Fig.S.4 was also made for intermediate structure assuming a distorted trigonal structure of solid mercury with a size of coherently diffracting crystallites under a strain parameter s=0.08±0.01. The likely BN peak located at 28.3° was considered as an excluded region. The lattice for the Solid Hg nanocrystals parameters were found to be a=0.290nm and α=71.4±0.7°.


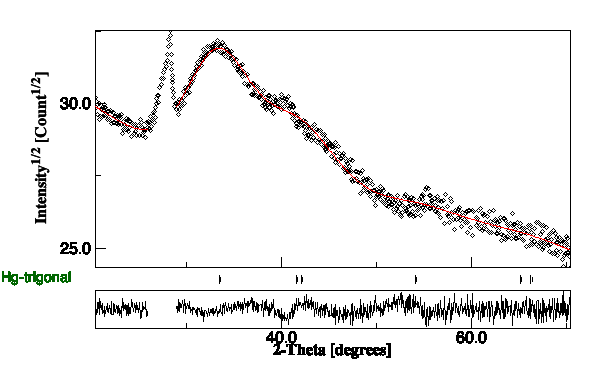


**Fig.S.4:** XRD spectrum of the Hg_1/20_-BN at N_2_ Temperature with its MAUD Rietveld treated profile.


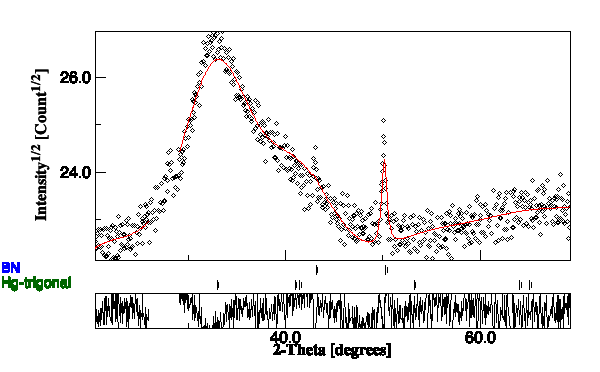


**Fig.S.5:** XRD spectrum of the Hg_1/4_-BN at Room Temperature with its MAUD Rietveld treated profile.

**S4. Modelling & Computational results**

**S4-1.Method of Calculation**

Two types of structures have been used for DFT calculations: the fcc cluster of Mercury and the rhombohedric Hg (101) on the hBN surface. The relative adsorption energy was obtained as:

${\Delta E}_{b}=E_{Hg-hBN}-E_{Hg}-E_{hBN}$ (1)

where, ${\Delta E}_{b}$ is the adsorption energy, $E_{Hg-hBN}$ is the energy of the given geometry containing the hBN surface and Hg surface (or cluster), $E_{Hg}$ is the energy of the rhombohedric Hg (101) surface (or cluster), and $E_{hBN}$ is the energy of the hBN surface. Also, the charge density difference of the system is defined as follows

${\Delta\rho}_{b}=\rho_{Hg-hBN}-\rho_{Hg}-\rho_{hBN}$ (2)

The adatom/B.N. system was modeled by considering one Hg atom on top of Boron in a B.N. supercell with periodic boundary conditions. Density functional theory (DFT) with the generalized gradient approximation (GGA) in the form of Perdew-Burke-Ernzerhof (PBE) implemented in the QUANTUM ESPRESSO package was performed with the ultra-soft pseudopotential [9] for all atoms. A $4\times1\times1$ Monkhorost-Pack mesh grid of k-points was used to sample the Brillouin zone [10]. The adsorption energy is about -0.22 eV, while the cohesive energy of Mercury is -0.67 eV [14]. Therefore, the small ratio of E_a_/E_c_ = 0.33 eV indicates the 3D growth will take place. Additionally, the difference between the cohesive energy of Mercury and its adsorption to the B.N. surface, E_c_-E_a_= -0.45 eV i,s relatively small and indicates the low thermal stability of formed mercury islands.

**S.4.1.1- Mercury cluster**

In the previous DFT calculations, the mercury cluster with fcc structure (Fig.5a) has been obtained as the stable structure with PBE, BP86, and PW91 functionals. Herein, the DFT calculations were performed using the Amsterdam Modelling suite of program (ADF). The generalized gradient approximation (GGA) of Perdew, Burke, and Ernzerhof (PBE) within the frozen core double-$\zeta$ polarized basis set (DZP) from the ADF basis set library. The influence of relativistic effects has been considered by comparing the non-relativistic (N.R.) and scalar-relativistic (S.R.) ZORA Hamiltonian . The dispersion interaction was carried via Grimme DFT-D3 corrections. Also, fcc cluster of Mercury on the hBN ribbon (Fig. 1b) was studied using the Quantum ESPRESSO [8]. There is 10 Å of vacuum in the y-z directions. The GGA-PBE method was utilized to describe the exchange-correlation functional together with the ultra-soft pseudopotential for all atoms. A $4\times1\times1$ Monkhorost-Pack mesh grid of k-points was used to sample the Brillouin zone [10]. The occupation of electronic states was determined using Gaussian smearing with the width of 0.01 eV, and the real space orbital cut-off of 8.4 Å was conducted.

**S.4.1.2- Mercury surface**

The experimental results reported the rhombohedric Hg (101) and Hg (003) surfaces formation at room temperature at nano-size (from 1.6 up to 3 nm) [1]. Therefore, the rhombohedric Hg (101) and Hg (003) with the size of ∼ 1.5 nm on top of the B.N. surface was considered in this work. The initial distance between the two surfaces was considered to be 3.6 Å which is the final distance between a cluster of Mercury on top of the hBN surface. The optimized geometries are shown in Fig.S.6. More accurately, the DFT calculations were performed using the Quantum ESPRESSO [8]. Here, the GGA-PBE method was utilized to describe the exchange-correlation functional together with the ultra-soft pseudopotential for all atoms. A $4\times1\times1$ Monkhorost-Pack mesh grid of k-points was used to sample the Brillouin zone . The occupation of electronic states was determined using Gaussian smearing with the width of 0.01 eV, and the real space orbital cut-off of 5.4 Å was conducted.

**S.4.1.3- Results**

The optimized geometry of the fcc cluster of Mercury on top of the one layer of the hBN surface is shown in Fig.5.a. The final distance between the mercury cluster and the surface is 3.6 Å, and the Hg-Hg bond length is between 3.53-3.60 Å. The energy decomposition analysis was carried out in view of obtaining the total binding energy and HOMO-LUMO gap. The results are summarized in Table 2. The interaction between the mercury cluster and the hBN surface is stabilized clearly by considering the dispersion corrections and increased from -0.23 to -0.86 eV. Therefore, -0.63 eV of the interaction energy is the dispersion correction contribution.

**Table 2.** The contribution of scalar relativistic effects and dispersion energy on the interaction of the mercury cluster with the hBN surface. NR and SR stand for non-relativistic and scalar relativistic contributions. D3 is the Grimme-D3 dispersion interaction.

| Functional | Relativistic effect | Binding energy (eV) | HOMO-LUMO energy gap (eV) | Hg-Hg  Bond (Å) |
| --- | --- | --- | --- | --- |
| PBE ^a^ | N.R. | -0.23 | -1.06 |  |
| PBE ^a^ | S.R. | -0.24 | -3.29 |  |
| PBE-D3 ^a^ | N.R. | -0.86 | -1.05 |  |
| PBE-D3 ^a^ | S.R. | -0.88 | -3.28 | 3.59 |
| PBE-D3^b^ | S.R. | -0.87 | -3.06 | 3.67 |

^a^ at the PBE/DZP level of theory.

^b^ at the PBE level of theory with plane-wave basis set.

Studies show that the relativistic effect has an important contribution in the 6s-6p and 6s-5d hybridization in the electronic structure of Hg. Also, the magnitude of mercury bonds highly affected by relativity. In order to investigate the change in the electronic structure of Mercury adsorbed on hBN surface near Fermi energy due to the relativistic effects, the molecular orbital energy levels are illustrated in Fig. S.7 without and with scalar relativistic contributions at the PBE-D3/DZP level of theory. As it is indicated from Fig. S.7, considering the relativistic effects change the band structures considerably. The bandgap increases from 1.05 to 3.1 eV due to scalar relativistic effects, which is in qualitative agreement with previous findings.

The fcc cluster of Mercury on the hBN surface was optimized at the PBE-D3 level of theory with the plane-wave basis set including the scalar relativistic effects, using the Quantum ESPRESSO package, shown in Fig 1b. The corresponding binding energy is just 0.01 eV less than our previous results at the PBE-D3/DZP level of theory which can be interpreted as the basis set correction contributions. The HOMO-LUMO gap energy, however decreases noticeably (~7 %) by degrading the level of theory, (see Table 2).

The interaction energies of Mercury (101) and (003) layers on the hBN surface performed by Quantum Espresso are presented in Table 3. The interaction energy of one layer of Hg (101) with the B.N. surface is around -3.03 eV, and the gap energy is -2.74 eV. Adding another layer of Hg

**Table 3.** The interaction energies (in eV) of Hg (101) surface on hBN surface at the PBE-D3 level of theory, including the relativistic effects and plane-wave basis set.

| Configuration | Binding energy (eV) | HOMO-LUMO energy gap (eV) | Hg-Hg  Bond (Å) |
| --- | --- | --- | --- |
| 1 layer of Hg (101) | -3.03814 | -2.7432 | 3.76 |
| 2 layers of Hg (101) | -3.04883 | -1.5252 |  |
| 1 layer of Hg (003) | -2.95 | -3.23 | 3.72 |

(101) to the configuration does not change the binding energy while decreasing the gap energy significantly. The optimized configurations are plotted in Fig.S.6. The final distance of the Hg (101) layer with the surface in Fig. S.6(a) is about 3.45 Å, and the length of Hg-Hg bonds varies between 3.78-3.95 Å (The mean value of the Hg-Hg bond is 3.76 Å). In Fig. S.6(b), the distance between the two Hg layers is about 3.57-3.84 Å, while the distance between the first Hg (101) layer and the hBN surface is almost 3.46-3.52 Å (The atoms in between are closer to the surface).

The interaction energy of the Hg (003) with the B.N. surface is around - 2.95 eV, and the gap energy is 3.23 eV. The mean length of the Hg-Hg bond is about 3.72 Å.

The charge difference between the mercury cluster, one layer of Hg (101), two layers of Hg (101), and the hBN surface are plotted in Fig S.7 . The iso-value of the charge difference is fixed to 0.0001 e a.u.^-3^. Yellow and blue colors indicate positive and negative levels correspond to accumulation and loss of electron charge density upon adsorption of Hg atoms. Changes in the charge density are most pronounced in the case of adsorption of Hg (101) and (003) surfaces, as the interaction energies are also indicating a stronger bond. Generally, the accumulation of charge is mostly around the mercury atoms.

**Fig.S.6:** (a) The fcc cluster of Mercury on hBN surface optimized at the PBE-D3/DZP level of theory using ADF. The final distance between the cluster and surface is about 3.6 Angstrom. The Hg-Hg bond is of order of 3.53 - 3.60 Angstrom, (b) The configuration of one cluster of Mercury on hBN surface optimized utilizing the plane-wave basis set with PBE-D3 functional using Quantum ESPRESSO. The distance between the Hg cluster and the next image of it is about 10.2 Angstrom. The final distance between the clusters and surface is about 3.45 Angstrom. The Hg-Hg bond is of order of 3.65 Angstrom.

**Fig. S.7:** (a) The optimized geometry of one Hg (101) surface on hBN surface (b) The optimized geometry of two Hg (101) layers on hBN surface (c) The optimized geometry of one Hg (003) surface on hBN surface. All optimizations were done at the PBE-D3/plane-wave level of theory including the scalar relativistic effects

| 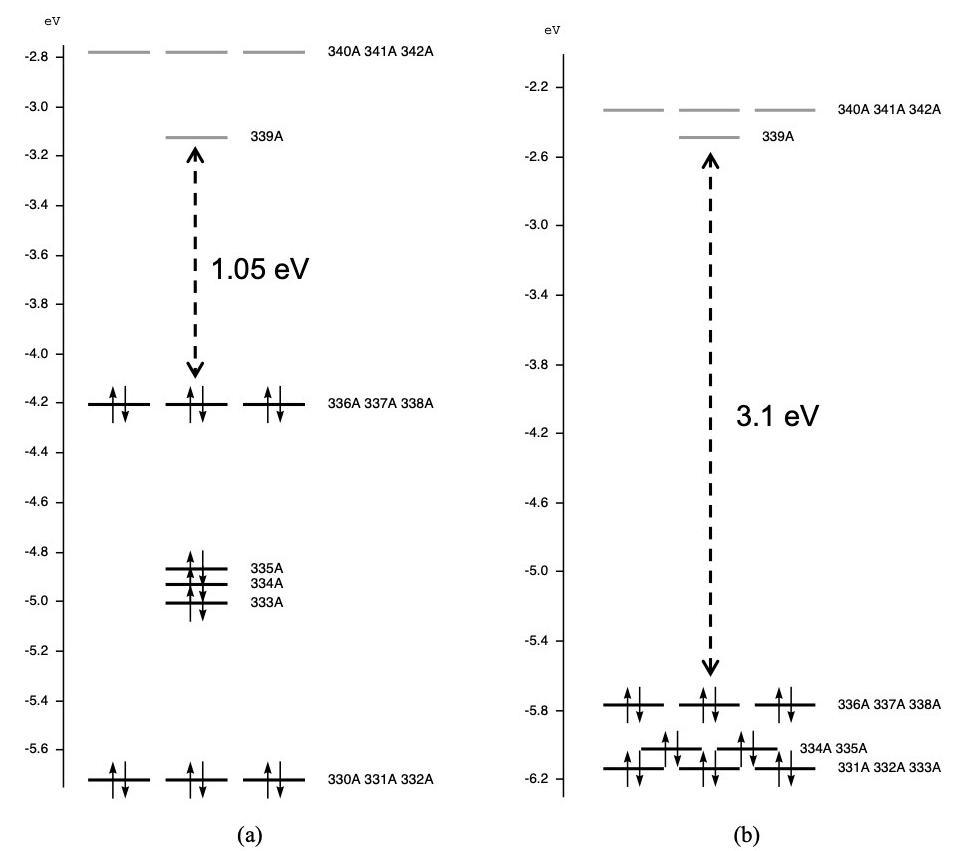 |
| --- |
| **Fig. S.8:** Molecular Orbital Energy level diagram around the Fermi level for one cluster of Hg on B.N. surface at the PBE-D3 level of theory, (a) without relativistic corrections (b) considering scalar relativistic corrections. Including the relativistic effects enlarges the bang gap significantly.   1. Lutterotti, L., Maud: a Rietveld analysis program designed for the internet and experiment   integration. *Acta Crystallogr. A* **2000,** *56*, s54. |
